# Convergent Processing of Both Positive and Negative Motivational Signals by the VTA Dopamine Neuronal Populations

**DOI:** 10.1371/journal.pone.0017047

**Published:** 2011-02-15

**Authors:** Dong V. Wang, Joe Z. Tsien

**Affiliations:** 1 Shanghai Institute of Brain Functional Genomics (Key Laboratory of MOE & STCSM), East China Normal University, Shanghai, China; 2 Brain and Behavior Discovery Institute and Department of Neurology, Georgia Health Sciences University, Augusta, Georgia, United States of America; Max-Planck-Institut für Neurobiologie, Germany

## Abstract

Dopamine neurons in the ventral tegmental area (VTA) have been traditionally studied for their roles in reward-related motivation or drug addiction. Here we study how the VTA dopamine neuron population may process fearful and negative experiences as well as reward information in freely behaving mice. Using multi-tetrode recording, we find that up to 89% of the putative dopamine neurons in the VTA exhibit significant activation in response to the conditioned tone that predict food reward, while the same dopamine neuron population also respond to the fearful experiences such as free fall and shake events. The majority of these VTA putative dopamine neurons exhibit suppression and offset-rebound excitation, whereas ∼25% of the recorded putative dopamine neurons show excitation by the fearful events. Importantly, VTA putative dopamine neurons exhibit parametric encoding properties: their firing change durations are proportional to the fearful event durations. In addition, we demonstrate that the contextual information is crucial for these neurons to respectively elicit positive or negative motivational responses by the same conditioned tone. Taken together, our findings suggest that VTA dopamine neurons may employ the convergent encoding strategy for processing both positive and negative experiences, intimately integrating with cues and environmental context.

## Introduction

Dopamine neurons in the ventral tegmental area (VTA) have been traditionally studied for their roles in reward-related motivation or drug addiction [1]–[3]. However, VTA dopamine neurons are also believed to be important for negative motivation [1]–[4]. In the literature, the role of the dopamine neuron in positive motivation has been well established and supported by many studies showing that reward (e.g., food, juice) and reward cues (conditioned stimuli) evoke a short-latency (50–110 ms) and short-duration (∼200 ms) burst activity of the dopamine neuron [5]–[9]. These dopamine neurons' responsiveness appears to encode a wide range of novel and reward-related events through a prediction error rule [5]–[9]. VTA dopamine activity has also been shown to play an essential role in drug addiction: almost all addictive drugs increase the synaptic dopamine level in the nucleus accumbens that receives extensive dopaminergic inputs from the VTA area [10]–[12].

The role of the VTA dopamine neuron in negative motivation has also been noted. A number of studies have found that aversive events (e.g., oral infusion of quinine or LiCl) or negative states (e.g., drug withdrawal) can alter dopamine concentrations in brain areas innervated by the VTA dopamine neurons [13]–[15]. In addition, disruption of dopamine transmission in the VTA downstream structures leads to impaired conditioning to aversive or fearful experiences [16], [17]. Moreover, dopamine levels can exhibit opposite functions in reinforcement on behavior: the lower dopamine level in the nucleus accumbens is believed to improve punishment- but impairs reward-based learning, while the higher dopamine level improves reward- but impairs punishment-based learning [18]. These above studies strongly suggest that VTA dopamine neurons also play an important role in processing negative motivational signals. However, the exact role of the VTA dopamine neuron in negative motivation is not fully clear.

On the other hand, recent studies find that dopamine neurons in the substantia nigra pars compacta (SNc) can respond to both reward (e.g., juice) and aversive stimuli (e.g., air puff) and two populations of SNc dopamine neurons may distinctly convey positive and negative motivational signals [9], [19]. However, concerns have been raised as to whether air puff to the skins, or conditioned cue predicting the occurrence of air puff, is truly aversive to monkeys as long as such activities are deemed not harmful [9]. Moreover, SNc dopamine neurons are known to process different aspects of information, and with distinct input-output neural circuitry as to the VTA [5]. Therefore, there is a strong interest in investigating whether and how the VTA dopamine neurons process negative experiences, and whether there are distinct dopamine neuron populations dedicating themselves to process positive and negative information.

To address these important questions, we employed multi-tetrode extracellular recording in freely behaving mice, and used two types of robust fearful events (free fall and shake) [20] as a way to study the role of the VTA neurons in processing negative motivational signals. We also trained mice to pair a neutral tone with subsequent food delivery, which allowed us to investigate how the same VTA dopamine neuron population may process positive motional signals. Moreover, because context information is such an integral part of many overall experiences, we asked whether and how environmental contexts may play a role in discriminating reward or aversive information. In this regard, we further carried out a set of experiments in which we trained mice to pair one single tone with both food reward and fearful event but in different contexts, which allowed us to determine how the conditioned VTA dopamine neural responses were intrinsically influenced by the environmental context. Our results suggest that VTA dopamine neurons may employ the convergent encoding strategy for processing both positive and negative experiences.

## Results

### Classification of putative dopamine neurons

**Figure 1:**
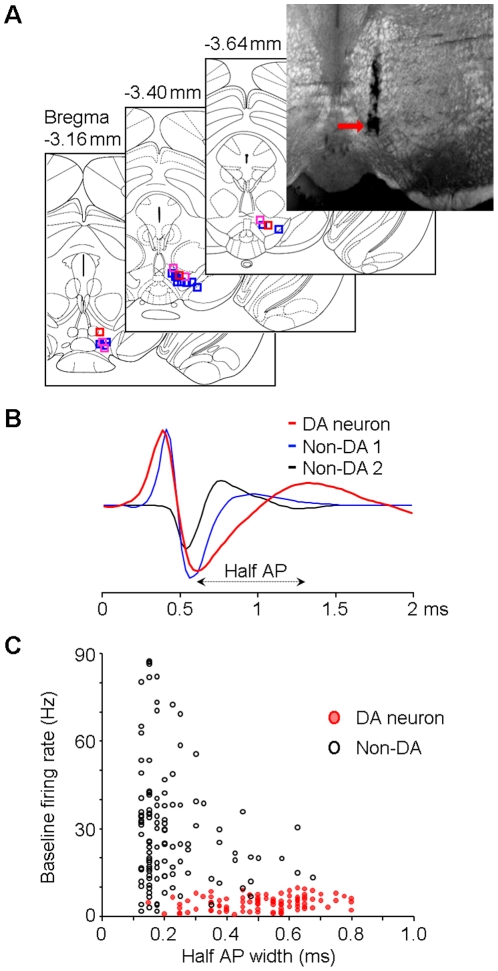
Muti-tetrode recording and VTA neuron classification. (**A**) Electrode array track shown on an example coronal brain section (top-right) and locations of the electrode array tips (from 21 mice) on atlas section diagrams [52]. Blue squares represent the locations where type-1/2 putative DA neurons were recorded; red squares represent the locations where type-3 neurons were recorded; purple squares represent the locations where both type-1/2 and type-3 neurons were recorded (see Figure 2 for the classification of the three types of putative DA neurons). (**B**) Examples of typically recorded spike waveforms for putative DA (red) and Non-DA (blue and black) neurons. Half AP width was measured from the trough to the following peak of the action potential. (**C**) Baseline firing rates and half AP widths of the classified DA (red) and Non-DA (black) neurons. DA, dopamine; Non-DA, non-dopamine; AP, action potential.

We implanted movable bundles of 8 tetrodes (32 channels) into the VTA of the right hemisphere of mice, and the recording electrodes' positions were confirmed by histology at the end of our experiment (Figure 1A). Data from 24 mice from which we recorded putative dopamine neurons were used in the current analyses. A total of 210 units with clear spike waveforms were recorded from these 24 mice (for examples of well-isolated units, see Figure S1). Of them, 96 units were classified as putative dopamine neurons based on their firing patterns (see Materials and Methods), and the other 114 units were thus classified as non-dopamine neurons. The classified putative dopamine neurons typically exhibited broad, tri-phasic action potentials (Figure 1B, red), although with variation, while non-dopamine neurons exhibited narrower tri-phasic or bi-phasic action potentials (Figure 1B, blue and black, respectively). Importantly, only neurons with low baseline firing rates (0.5–10 Hz; Figure 1C), relatively long inter-spike interval (>4 ms) and regular firing pattern were classified as putative dopamine neurons. In contrast, classified non-dopamine neurons typically showed higher baseline firing rate (>10 Hz; Figure 1C) and/or significant modulation in firing rate during movement, relative to quiet wakefulness [21]–[23].

### Three types of fear-responsive VTA putative dopamine neurons

**Figure 2:**
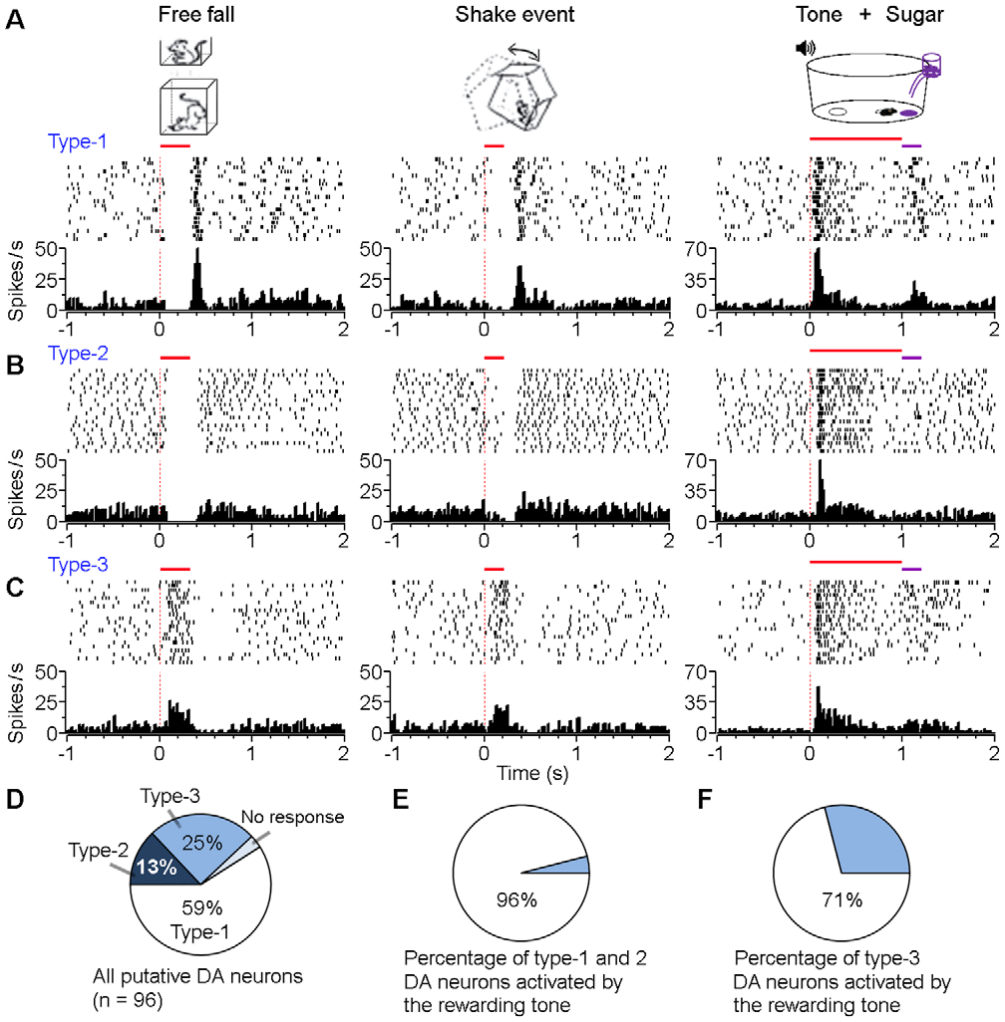
Three types of VTA putative dopamine (DA) neurons. (**A-C**) Peri-event rasters (1-20 trials, from top to bottom) and histograms of three examples of VTA putative dopamine neurons (A: type-1, B: type-2, and C: type-3) in response to free fall (left panels), shake (middle panels), and the conditioned tone that reliably predicted sugar pellet delivery (right panels). (**D**) Percentages of different types of putative DA neurons. (**E, F**) Percentages of fear-suppressed (E: type-1 and 2) and fear-excited (F: type-3) putative DA neurons that were significantly activated by the conditioned tone that reliably predicted sugar pellet delivery. Free fall, 30 cm high; Shake, 0.2 sec; Tone, 5 kHz, 1 sec.

We used two types of robust fearful events (free fall and shake) for examining how the VTA neurons might respond to negative experiences [20]. After mice recovered from the surgeries and stable recordings were achieved (usually 1∼2 weeks post-surgery), we began the experiments. Each mouse was placed into a free-fall chamber or shake chamber, where about 20 trials of free fall or shake events were given each session with an interval of 1–2 min between trials (Figure 2A). The interval between sessions is typically 1–2 hours. We always monitored the stability of recorded units by examining the spike waveform shapes, baseline firing status, and spike cluster distributions before and after the events as well as through the entire experiments. We assessed that there was no temporary loss of units during the two fearful events by examining simultaneously recorded units (e.g., two units recorded from the same tetrode showing opposite firing changes) (Figure S2). We also ensured that no artificial electrical or mechanical noises were included in the recorded data by assessing the waveforms right before, during and after the fearful events (Figure S3). Overall, these putative dopamine neurons (n = 96) were largely divided into three major types based on their response properties to the two fearful events: type-1 (59%, 57/96), type-2 (13%, 12/96) and type-3 (25%, 24/96).

Type-1 VTA putative dopamine neurons showed not only significant suppression of their firing in responses to both the free fall and shake events (Figure 2A, left and middle panels) (*P*<0.05, Wilcoxon signed-rank test), but also a strong offset-rebound excitation at the termination of both events. We defined the rebound excitation as the offset peak firing rate (smoothed with a Gaussian filter) to be at least two times higher than the baseline firing rate and with z-scores larger than 2. Such rebound excitation may signal the safety at the end of fearful events or a motivation by such events. We then asked whether these type-1 dopamine neurons were responsive to reward signals. By repeatedly pairing a neutral tone with the subsequent delivery of a sugar pellet, we found that these neurons also significantly increased their firing to the conditioned tone that reliably predicted reward (Figure 2A, right panel). Therefore, these type-1 dopamine neurons were responsive to both reward and negative signals.

Type-2 VTA putative dopamine neurons showed significant suppression (*P*<0.05, Wilcoxon signed-rank test) during free fall or shake, but they did not have rebound activation after these events were terminated (z-scores <2) (Figure 2B, left and middle panels). Similar to the type-1 putative dopamine neurons, these type-2 neurons increased their firing significantly to the conditioned tone that reliably predicted reward (Figure 2B, right panel). Thus, both type-1 and type-2 dopamine neurons exhibit bidirectional modulation by the negative and positive events, that is, they decrease their firing to the fearful events while increasing their firing to the reward signals.

Intriguingly, we also recorded a third type of dopaminergic-like neurons, which shared more similarity with type-1/2 putative dopamine neurons rather than non-dopamine neurons. These type-3 neurons (about 25% of all recorded putative dopamine neurons) increased their firing to both free fall and shake events (Figure 2C, left and middle panels) (*P*<0.05, Wilcoxon signed-rank test). Their increased firing was typically followed by an offset suppression. Moreover, these type-3 dopamine neurons can also increase their firing in response to the conditioned tone that predicted reward (Figure 2C, right panel). These type-3 neurons, which increased their firing to both positive and negative events, are quite distinct from type-1 and type-2 dopamine neurons. This strongly suggests the diversity of the VTA dopamine neuron population [24], [25].

Overall, type-1 and type-2 neurons constitute a majority (72%) of the recorded VTA putative dopamine neuronal population, whereas type-3 neurons constitute about 25%, with the remaining putative dopamine neurons (3%) not responsive to the fearful events (Figure 2D). Moreover, our analyses suggest that all of these neurons' responses to the negative events tend to be directionally uniform (45 neurons tested for both free fall and shake events), that is, neurons suppressed (or activated) by the free fall event were always suppressed (or activated) by other fearful events, such as the shake event, and vice versa. Out of the fear-suppressed dopamine neurons (type-1 and type-2) that we examined for their responsiveness to the reward signals, 96% of them (44/46) showed significant activation by the rewarding tone (Figure 2E) (*P*<0.05, Wilcoxon signed-rank test). This clearly shows that the vast majority of type-1 and type-2 VTA dopamine neurons are capable of bi-directionally responding to both positive and negative events, that is, they show excitation by reward information while suppression by fearful experiences. On the other hand, about 71% of the type-3 putative dopamine neurons (12/17) that were activated by fearful events can also be activated by the reward signals (Figure 2F) (*P*<0.05, Wilcoxon signed-rank test). This strongly suggests that the fearful events, not just reward, can excite some of the VTA putative dopamine neurons.

### Firing patterns and pharmacology characterizations

**Figure 3:**
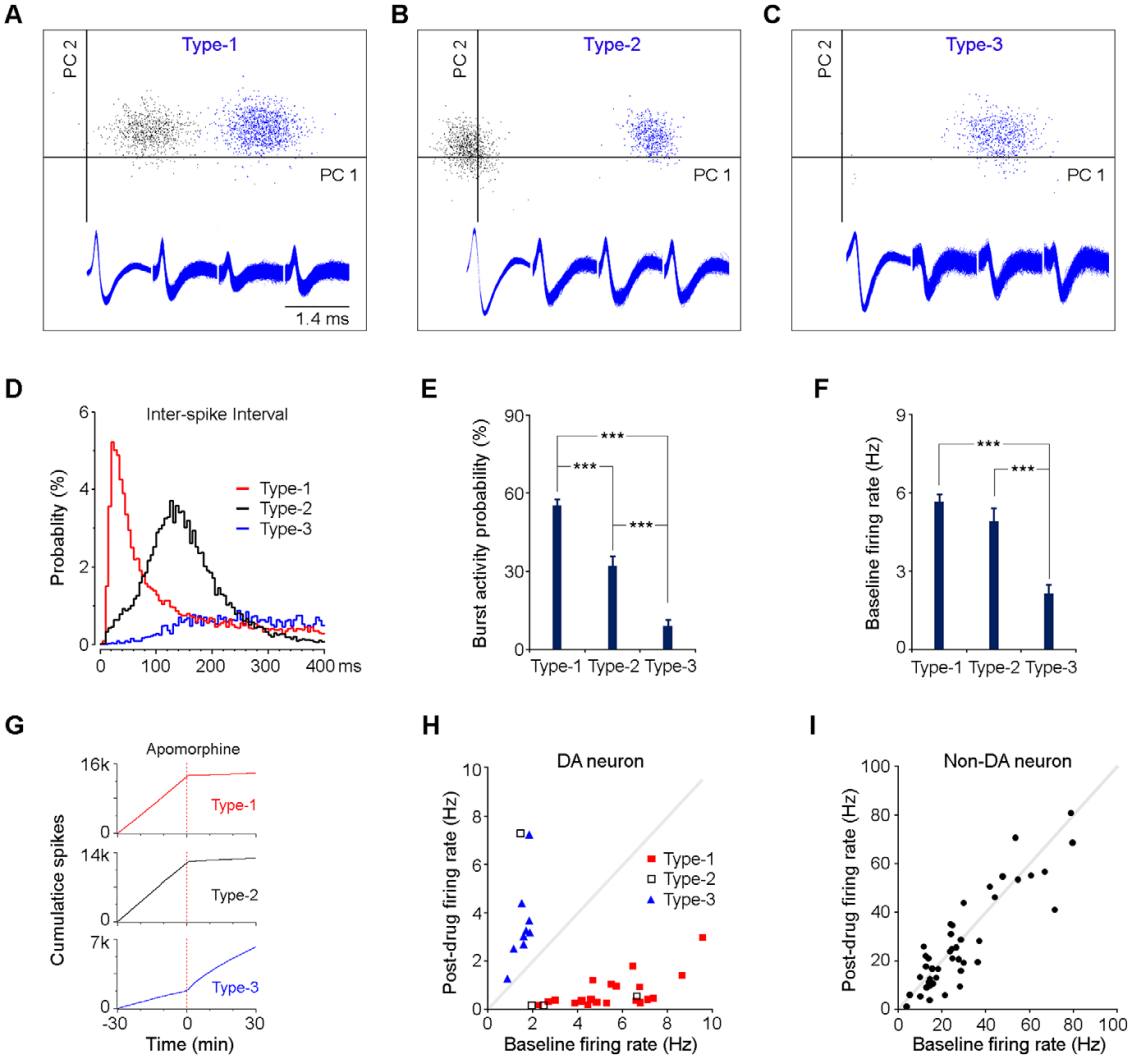
Firing patterns and pharmacology characterizations. (**A–C**) Three examples of tetrode-recorded putative dopamine neurons (type-1, type-2, and type-3) and their representative spike waveforms. PC1 and PC2 represent the first and second principal components in the principal component analysis, respectively. Blue dots represent individual spikes for the isolated dopamine neurons; black dots indicate individual spikes for other unsorted VTA neurons. (**D**) Inter-spike intervals of three examples of putative dopamine neurons (type-1, type-2, and type-3). (**E**) Percentages of burst firing for the three types of putative dopamine neurons. Error bars, s.e.m.; ***P<0.001, Student's *t*-test. (**F**) Baseline firing rates of the three types of putative dopamine neurons. Error bars, s.e.m.; ***P<0.001, Student's *t*-test. (**G**) Cumulative spike activity of thee examples of putative dopamine neurons (type-1, type-2, and type-3) in response to the dopamine receptor agonist apomorphine. It was noted that the type-1 and type-3 putative dopamine neurons were recorded simultaneously from one tetrode. (**H** and **I**) Baseline and post-drug firing rates of putative dopamine (H) and non-dopamine (I) neurons. Mice were injected with the dopamine receptor agonist apomorphine (1 mg/kg, i.p.) and the firing rates were averaged 30 min before and 30 min after apomorphine injection.

Despite their similarities in firing pattern and spike waveforms of the three types of putative dopamine neurons (e.g., Figure 3A–C), we did notice some differences among them. First, type-3 dopaminergic-like neurons exhibited a significantly lower probability (9±2.3%, mean ± s.e.m.) of burst firing compared with type-1 (55.2±2.5%) or type-2 (32.0±3.8%) putative dopamine neurons (Figure 3D and E). Second, type-3 neurons showed a much lower baseline firing rate (2.15±0.33 Hz, mean ± s.e.m.; n = 24) compared with type-1 (5.66±0.27 Hz; n = 57) or type-2 (4.92±0.49 Hz; n = 12) neurons (Figure 3F).

We also injected the mice with the dopamine receptor agonists apomorphine (1 mg/kg, i.p.) and/or quinpirole (1 mg/kg, i.p.) which have been mainly shown to inhibit the activity of the dopamine neuron [6], [8], [24], [25]. A total of 77 VTA neurons (including 33 classified putative dopamine neurons and 44 non-dopamine neurons) were tested with the dopamine receptor agonists. Our pharmacological results revealed that the vast majority (96%; 23/24) of type-1 and type-2 putative dopamine neurons were significantly suppressed, while surprisingly the type-3 neurons (n = 9) otherwise showed excitation by apomorphine (Figure 3H). In addition, 4 classified putative dopamine neurons were tested with both apomorphine and quinpirole (on different days). These 4 putative dopamine neurons exhibited similar responses to apomorphine and quinpirole: neurons (n = 2) suppressed by apomorphine were also suppressed by quinpirole; neurons (n = 2) activated by apomorphine were also activated by quinpirole. In contrast, VTA non-dopamine neurons (n = 44) showed very limited or no changes in firing rate after the injection of apomorphine or quinpirole (Figure 3I).

### Responses of VTA putative dopamine neurons to different durations and intensities of fearful events

**Figure 4:**
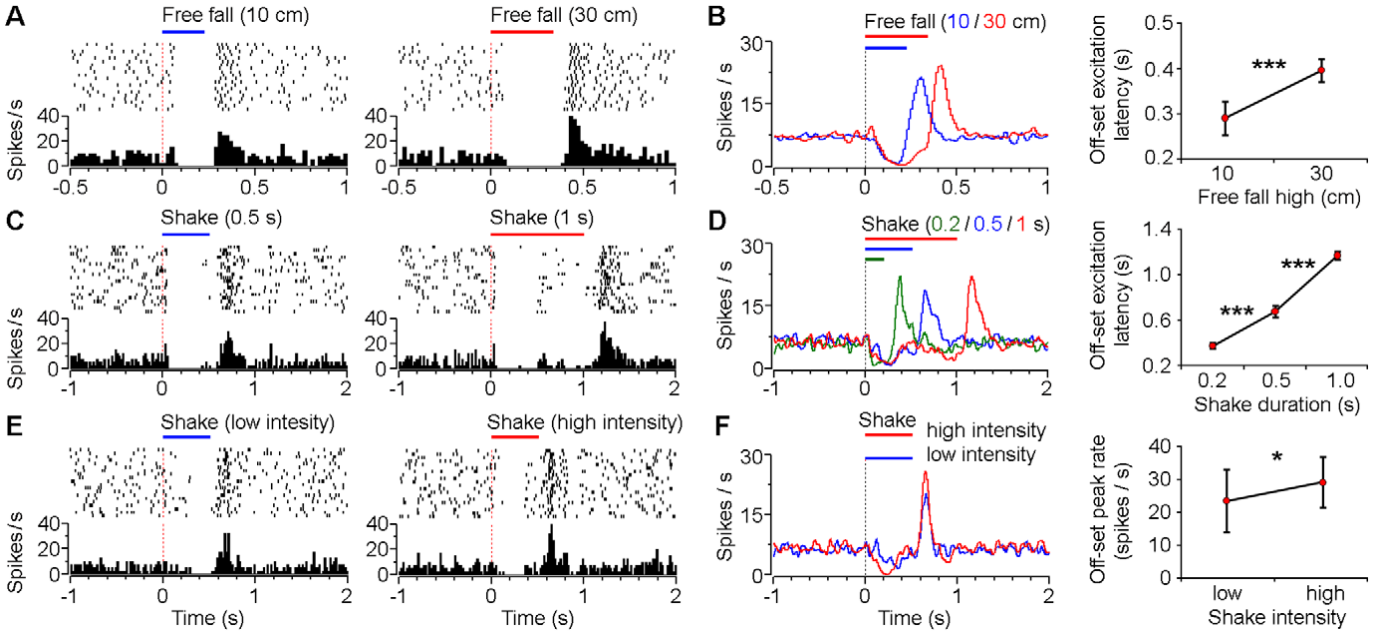
Responses of VTA type-1 putative dopamine neurons to different durations and intensities of fearful events. (**A**) Peri-event rasters (1–20 trials) and histograms of one example type-1 neuron in response to 10 cm (left) and 30 cm (right) free fall events. (**B**) Smoothed population average peri-event histograms (left) and offset excitation latencies (right) of type-1 neurons in response to 10 cm (blue line; n = 15) and 30 cm (red line; n = 20) free fall events. (**C**) Peri-event rasters and histograms of another type-1 neuron in response to 0.5 sec (left) and 1 sec (right) shake events. (**D**) Smoothed population average peri-event histograms (left) and offset excitation latencies (right) of type-1 neurons in response to 0.2 sec (green line; n = 13), 0.5 sec (blue line; n = 20), and 1 sec (red line; n = 14) shake events. (**E**) Peri-event rasters and histograms of another type-1 neuron in response to low-(left) and high-intensity (right) shake events. (**F**) Smoothed population average peri-event histograms (left) and offset excitation peak firing rates (right) of type-1 neurons in response to low- (blue line; n = 9) and high-intensity (red line; n = 9) shake events. Error bars, s.d.; *P<0.05, ***P<10^-8^, Student's *t*-test.

To further understand the encoding properties of the VTA dopamine neurons for fearful events, we conducted a set of parametric experiments. Different heights of free fall (10 and 30 cm) and different durations of shake (0.2, 0.5 and 1 sec) were performed in random orders during the recording experiments. We found that VTA dopamine neurons exhibited temporal dynamic activity changes that were proportional to the durations of the fearful events. As shown in Figure 4A, type-1 putative dopamine neurons exhibited duration-dependent suppression during free fall events (10 cm vs. 30 cm high). Population analysis revealed that, in response to 10 and 30 cm free fall events (Figure 4B), the average offset excitation latencies (latency of the smoothed offset peak firing rate) of type-1 putative dopamine neurons were 293±38 ms (mean ± s.d., n = 15) and 398±28 ms (n = 20), respectively (*P*<0.001, Student's *t*-test). These results suggest that responses of the type-1 putative dopamine neurons correlate with the duration of the fearful events (Figure 4B, right panel). It was noted that the offset peak firing rate was slightly higher during the 30 cm free fall event (30.9±6.6 Hz; mean ± s.d.) compared with the 10 cm event (26.3±5.9 Hz) (*P* = 0.04, Student's *t*-test), suggesting that responses of type-1 VTA dopamine neurons to negative may also reflect, to less a degree, the intensity of the free fall events.

Similarly, these type-1 neurons showed duration-dependent response properties to the shake events (Figure 4C and D). The average offset excitation latencies were 374±25 ms (mean ± s.d., n = 13), 672±52 ms (n = 20) and 1169±35 ms (n = 14) for shake events that lasted for 0.2, 0.5, and 1 sec, respectively (*P*<0.001, one-way ANOVA). Follow-up Student's *t*-tests showed highly significant differences for each comparison (Figure 4D, right panel). However, there were no significant differences in the offset peak firing rates across different durations of the shake events (*P*>0.05; one-way ANOVA). We also varied the intensity of the shake event: type-1 neurons exhibited slightly higher offset excitation peak by the high-intensity shake events compared with the low-intensity one (Figure 4E and F; 29.1±7.7 vs. 23.5±9.5 Hz, mean ± s.d.). These above results suggest that responses of VTA type-1 putative dopamine neurons correlate with the duration of fearful events, and to less a degree, the intensity of fearful events.

**Figure 5:**
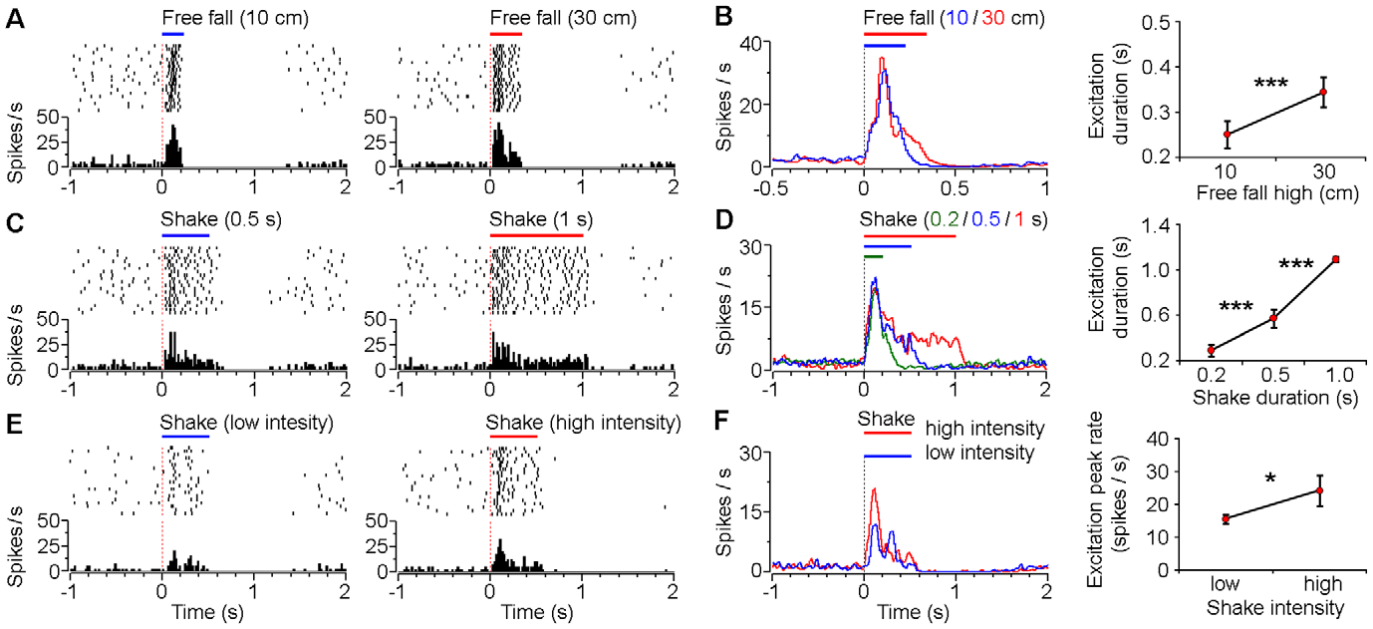
Responses of VTA type-3 dopaminegic-like neurons to different durations and intensities of fearful events. (**A**) Peri-event rasters (1-20 trials) and histograms of one example type-3 neuron in response to 10 cm (left) and 30 cm (right) free fall events. (**B**) Smoothed population average peri-event histograms (left) and offset excitation latencies (right) of type-3 neurons in response to 10 cm (blue line; n = 8) and 30 cm (red line; n = 10) free fall events. (**C**) Peri-event rasters and histograms of the same neuron (as shown in A) in response to 0.5 sec (left) and 1 sec (right) shake events. (**D**) Smoothed population average peri-event histograms (left) and offset excitation latencies (right) of type-3 neurons in response to 0.2 sec (green line; n = 10), 0.5 sec (blue line; n = 9), and 1 sec (red line; n = 7) shake events. (**E**) Peri-event rasters and histograms of another type-3 neuron in response to low- (left) and high-intensity (right) shake events. (**F**) Smoothed population average peri-event histograms (left) and offset excitation peak firing rates (right) of type-3 neurons in response to low- (blue line; n = 5) and high-intensity (red line; n = 5) shake events. Error bars, s.d.; *P<0.05, ***P<10^-5^, Student's *t*-test.

Moreover, the excitation duration of type-3 dopaminergic-like neurons also correlated with the duration of fearful events. In response to 10 and 30 cm free fall events (Figure 5A and B), the excitation durations were 251±29 ms (mean ± s.d., n = 8), and 345±33 ms (n = 10), respectively (*P*<0.001, Student's *t*-test). In response to 0.2, 0.5 and 1 sec shake events (Figure 5C and D), the excitation durations of type-3 neurons were 294±53 ms (n = 10), 573±80 ms (n = 9) and 1091±23 ms (n = 7), respectively (*P*<0.001, one-way ANOVA). Follow-up Student's *t*-test showed highly significant differences for each comparison (Figure 5D, right panel). In response to different intensities of shake events, type-3 neurons exhibited higher excitation peak by the high-intensity shake events compared with the low-intensity one (Figure 5E and F; 24.2±4.6 vs. 15.5±1.3 Hz, mean ± s.d.).

Together, these results suggest that the temporal dynamic changes in the firing of VTA putative dopamine neurons correlated well with the stimulus durations of the fearful events, with suppressed firing for type-1 and type-2 neurons and increased firing for type-3 neurons. Their firing changes may also correlate with the stimulus intensities of the fearful events, but to much less a degree.

### Integral encoding of events and contexts

The brain typically processes episodic experiences in environmental contexts, and this is also true for addictive behaviors. Contextual information has been suggested to be important for the responsiveness of dopamine neurons to reward predicting cues [26]. We asked whether environmental context played a role in encoding negative events, and more importantly, how the VTA dopamine neurons would respond to the same conditioned cue but co-linked to distinct contexts that would predict opposite outcome (e.g., reward vs. aversive stimuli).

**Figure 6:**
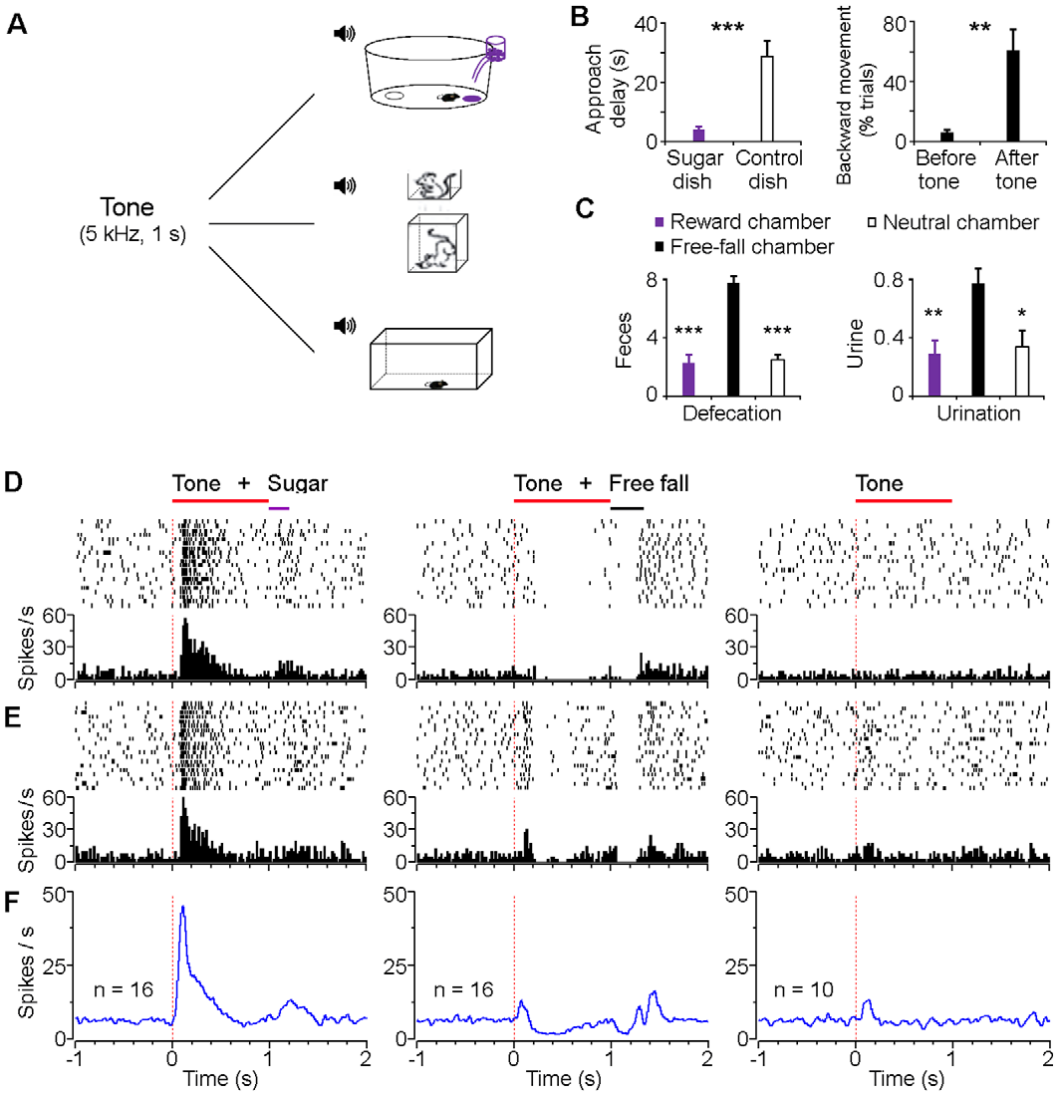
Bi-directional encoding of positive and negative signals via the same conditioned tone in different contexts. (**A**) Schematic of experimental paradigm for bidirectional conditioning. One tone (5 kHz, 1 sec) was used throughout: it predicted sugar pellet delivery in the reward chamber (top); it predicted free fall event in the free-fall chamber (middle); and it did not predict anything in the neutral chamber (bottom). (**B**) Left, delay of dish approach after onset of the conditioned tone that predicted sugar delivery. Right, mice showed significant increased backward movement after onset of the conditioned tone that predicted free fall event. (**C**) Aversion-like behaviors (frequent defecation and urination) elicited in the free-fall chamber compared with the reward or neutral chamber. Error bars, s.e.m.; n = 10; *P<0.05, **P<0.01, ***P<0.001, Student's *t*-test. (**D, E**) Peri-event rasters (1–20 trials) and histograms of two examples of VTA putative dopamine neurons in response to the same conditioned tone that predicted sugar pellet delivery (left), that predicted free fall event (middle), and that did not predict anything (right), with an interval of 1–2 h between sessions. (**F**) Smoothed population average peri-event histograms of fear-suppressed (type-1 and 2) putative dopamine neurons in response to the same conditioned tone that predicted sugar pellet (left panel; n = 16), that predicted free fall event (middle panel; the same 16 neurons as shown in the left panel), and that did not predict anything (right panel; n = 10). Free fall, 30 cm high.

Thus, we conducted another set of experiments in which mice were subjected to bidirectional conditioning (both reward and aversive conditioning). We used one neutral tone as the conditioned stimulus (CS) to pair with distinct unconditioned stimuli (US, either sugar pellet or free fall) in different environments (Figure 6A). We subjected the mice to Pavlovian conditioning for one week during which mice received ∼200 CS/US pairings for both reward and aversive conditioning (see Materials and Methods). After training, mice approached the sugar pellet receptacle quickly, typically in 3–10 sec (4.3 sec on average) after onset of the conditioned tone, but with no apparent approaching to the control dish that did not receive sugar pellets, indicating the effectiveness and specificity of the associative reward learning (Figure 6B, left panel). On the other hand, in response to the conditioned tone that predicted a free fall event in the free fall chamber, mice showed significant increased backward movement upon hearing the conditioned tone (Figure 6B, right panel), which may reflect an animal's avoidance or defensive behavior [27]. The heighted fear/anxiety responses in these mice were also evident from the increased defecation and urination in the free fall chamber compared with the reward or neutral chambers (Figure 6C).

Neuronal activity recordings in these conditioned mice (after 1-week training) revealed that VTA putative dopamine neurons responded significantly to the conditioned tone that predicted a sugar pellet in the reward chamber (Figure 6D, left panel). Interestingly, the same VTA neurons also responded reliably to the same conditioned tone when it predicted free fall in the free fall chamber (Figure 6D, middle panel). When the same conditioned tone was delivered to mice in a neutral chamber that was not associated with any event, it did not produce significant changes in firing (Figure 6D, right panel).

In total, we recorded 16 fear-suppressed (type-1 and type-2) dopamine neurons from the mice that were subjected to the bidirectional conditioning protocol. All of these neurons exhibited a significant increase in firing rates after onset of the conditioned tone that reliably predicted the sugar pellet (Figure 6D–F, left panels) (*P*<0.001, Wilcoxon signed-rank test). In response to the same tone that predicted the free fall event, half of the neurons (8/16) showed a significant decrease in firing rate (Figure 6D, middle panel) (*P*<0.05, Wilcoxon signed-rank test), while the other half (8/16) showed a brief immediate activation peak (at least two times higher than the baseline firing rate and with z-scores larger than 2) followed by a significant suppression (Figure 6E, middle panel) (*P*<0.05, Wilcoxon signed-rank test). In response to the same tone represented in a neutral chamber, there was very limited or no changes in firing (Figure 6D–F, right panels). These results suggest that type-1 and type-2 VTA putative dopamine neurons can bi-directionally encode the integrated positive and negative signals (conditioned tone and context information combined) by increasing and decreasing their firings, respectively.

**Figure 7:**
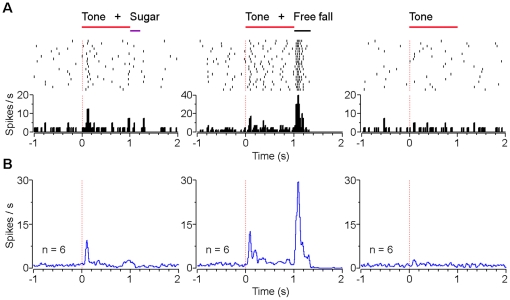
Responses of type-3 dopaminergic-like neurons to positive and negative signals via the same conditioned tone in different contexts. (**A**) Peri-event rasters (1–20 trials) and histograms of an example type-3 neuron in response to the same conditioned tone that predicted sugar pellet delivery (left), that predicted free fall event (middle), and that did not predict anything in the neutral chamber (right). (**B**) Smoothed population average peri-event histograms of type-3 neurons (n = 6) in response to the same conditioned tone that predicted sugar pellet delivery (left), that predicted free fall event (middle), and that did not predict anything (right). Free fall, 30 cm high.

The importance of contexts in producing distinct conditioned responses was also evident in type-3 dopaminergic-like neurons. As an example, the type-3 neuron responded significantly to the conditioned tone that was associated with a sugar pellet in the reward chamber (Figure 7A, left panel) or free fall in the free fall chamber (Figure 7A, middle panel). On the other hand, it did not show any change in firing rate when the tone was played in the neutral chamber (Figure 7A, right panel). Population analysis again, confirmed that these type-3 neurons increased their firing to the same conditioned tone in the reward and free fall chambers (Figure 7B, left and middle panels), but not in the neutral chamber (Figure 7B, right panel) (P<0.05, Student's *t*-test). Collectively, the above contextual experiments suggest that information represented at the VTA dopamine neuron level is highly processed and richly integrated to encode a given set of positive or negative motivational events associated with environmental contexts.

### Response onset latency of VTA dopamine neurons

We next set out to examine the response onset latency of the putative dopamine neurons to both the reward and fearful events. Peri-event histograms of 10 and 30 cm free fall events and peri-event histograms of 0.2, 0.5, and 1 sec shake events were combined for individual dopamine neurons for the calculation of the response onset latency. Response onset latency was determined by first obtaining the mean firing rate (mean) and standard deviation (s.d.) from the 1000 bins (bin  = 10 ms) immediately before the stimulus onset. Response latency was taken to be the time corresponding to the first bin of at least three consecutive bins with Z-scores ≥2 after onset of the stimulus. Due to the low baseline firing rate of the dopamine neuron, peri-event histograms (bin  = 10 ms) were smoothed with a Gaussian filter (filter width  = 3 bins) for the calculation of response onset latency of suppression (response onset latencies of type-1 and type-2 neurons to free fall, shake and the aversive CS).

**Figure 8:**
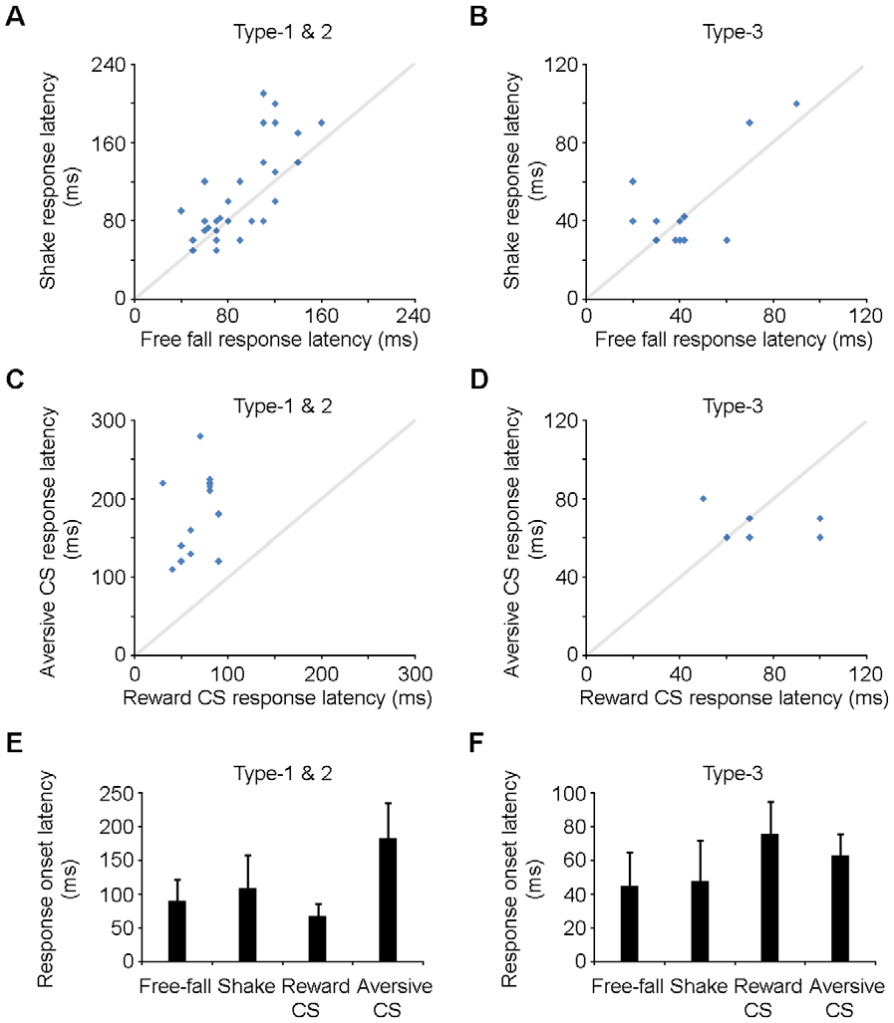
Response onset latencies of the VTA putative dopamine neurons. (**A**) Response onset latencies of individual type-1 and 2 dopamine neurons to free fall and shake events. (**B**) Response onset latencies of individual type-3 dopamine neurons to free fall and shake events. (**C**) Response onset latencies of individual type-1 and 2 dopamine neurons to the reward CS that predicted sugar pellet and the aversive CS that predicted free fall. (**D**) Response onset latencies of individual type-3 dopamine neurons to the reward CS that predicted sugar pellet and the aversive CS that predicted free fall. (**E**) Population average response onset latencies of type-1 and 2 dopamine neurons (from the same data as shown in A and C) and **(F)** type-3 neurons (from the same data as shown in B and D). Response onset latencies for type-1/2 neurons to free fall, shake and aversive CS correspond to the latencies of suppression; while the others correspond to the latencies of activation. Error bars, s.d.

Our results showed that type-1 and type-2 putative dopamine neurons exhibited similar response onset latencies to free fall and shake events (90.6±31.3 ms vs. 108.4±48.6 ms; mean ± s.d.) (Figure 8A and E). Type-3 dopaminergic-like neurons also exhibited similar response onset latencies to the two fearful events (43.5±20.6 ms vs. 46.8±24.2 ms), as well as to the two conditioned stimuli (75.7±19.0 ms vs. 62.9±12.5 ms) (Figure 8B, D and F). On the other hand, type-1 and type-2 neurons exhibited much longer response onset latency (of suppression) to the aversive CS in compare with the response onset latency (of activation) to the reward CS (181.6±51.9 ms vs. 67.1±19.0 ms) (Figure 8C and E). Overall, the response onset latency of suppression was generally longer than the response onset latency of activation for any comparison (Figure 8E and F).

### Synchrony among unique sets of VTA dopamine neurons

**Figure 9:**
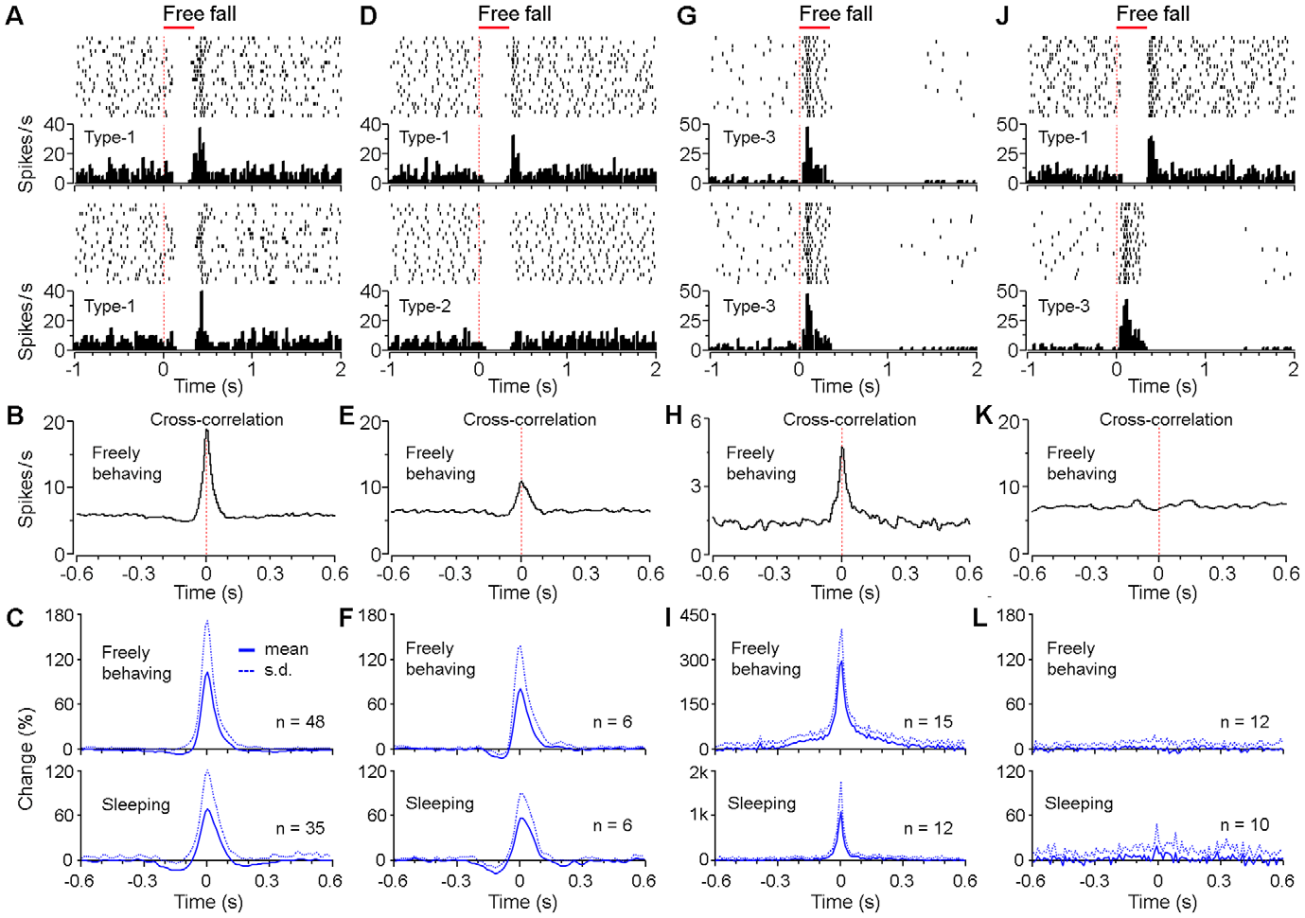
Synchrony among unique sets of VTA putative dopamine neurons. (**A**) Peri-event rasters (1–20 trials) and histograms of two simultaneously recorded type-1 neurons in response to free fall event, and (**B**) the cross-correlogram between these two neurons when the mouse was freely behaving. (**C**) The averaged cross-correlograms between simultaneously recorded type-1 neurons (48 pairs during freely behaving, and 35 pairs during sleep). (**D**) Peri-event rasters and histograms of two simultaneously recorded type-1 and type-2 neurons in response to free fall event, and (**E**) the cross-correlogram between these two neurons during freely behaving. (**F**) The averaged cross-correlograms between simultaneously recorded type-1 and type-2 neurons (6 pairs during both freely behaving and sleep). (**G**) Peri-event rasters and histograms of two simultaneously recorded type-3 neurons in response to free fall event, and (**H**) the cross-correlogram between these two neurons during freely behaving. (**I**) The averaged cross-correlograms between simultaneously recorded type-3 neurons (15 pairs during freely behaving, and 12 pairs during sleep). (**J**) Peri-event rasters and histograms of two type-1 and type-3 neurons (simultaneously recorded from one tetrode) in response to free fall event, and (**K**) the cross-correlogram between these two neurons during freely behaving. (**L**) The averaged cross-correlograms between simultaneously recorded type-1 and type-3 neurons (12 pairs during freely behaving, and 10 pairs during sleep). Free fall, 30 cm high.

Since dopamine levels in the target areas have been often linked to various cognitive outcomes, it has long been hypothesized that synchronized firing of dopamine neurons may represent a neural mechanism for implementing this neural chemical strategy [28], [29]. This notion is supported by studies showing that subsets of dopamine neurons in the substantia nigra pars compacta (SNc) exhibited spontaneous synchronized activity [24], [30]. By using multi-tetrode recording in our experiments, we had an opportunity to examine the dynamic correlations among the simultaneously recorded putative dopamine neurons in the VTA (with up to five putative dopamine neurons recorded simultaneously). Our analyses revealed that the vast majority of the putative dopamine neurons showed spontaneously synchronized firing, irrespective of the animal's sleep-awake cycle (Figure 9). As an example, cross-correlation of two simultaneously recorded type-1 putative dopamine neurons were highly significant (Figure 9A and B). From the analysis of the pooled datasets, by and large, the vast majority (83%; 48/58 pairs) of simultaneously recorded type-1 neurons showed significant synchronization (peak z-score >11) in a time window of about 100 ms regardless of whether mice were freely behaving or sleeping (Figure 9C). Similarly, there was also a significant synchronization between simultaneously recorded type-1 and type-2 putative dopamine neurons (Figure 9D–F). Out of the simultaneously recorded type-1 and type-2 dopamine neuron pairs, 75% (6/8) of them showed significant synchronization when mice were either freely behaving or sleeping (Figure 9F).

In addition, significant synchronization was also observed within the type-3 putative dopamine neuron population (Figure 9G–I). Out of the simultaneously recorded type-3 dopamine neuron pairs, 79% (15/19) of them showed significant synchronization (Figure 9I). On the other hand, when the simultaneously recorded type-1 and type-3 neurons, or type-2 and type-3 dopamine neurons (n = 12 pairs) were calculated for their cross-correlations, it did not reveal any significant synchronization (Figure 9J–L). Together, the synchronized activity among fear-suppressed putative dopamine neurons (type-1 and type-2), as well as among the fear-excited type-3 neurons suggests that different subpopulations of putative dopamine neurons may receive distinct inputs from separate brain areas and are integrated with distinct networks [25], [31], [32].

## Discussion

Our above ensemble recordings and analyses have provided a set of evidence for the role of the dopamine neurons in processing both positive and negative experiences. We found that VTA dopamine neurons exhibited diverse response properties and the vast majority of the putative dopamine neurons respond to both reward and fearful stimuli. This convergent encoding strategy by the VTA dopamine neurons is interesting in light of a highly cited study in awake monkeys which shows that dopamine neurons preferentially respond to stimuli with appetitive rather than aversive motivational value [33]. Aversive stimulus such as air puff used in this study is a rather mild stimulus in compare with the two fearful events used in our experiment. Some researchers have suggested that aversive stimulus like air puff may not exhibit negative value because monkeys can learn to blink or close their eyes to the conditioned stimulus to avoid the aversive stimulus [9], [34]. On the other hand, more recent studies in awake monkeys shows the existence of different types of dopamine neurons in the substantia nigra pars compacta **(**SNc) for conveying both positive and negative signals [5], [9], [19]. Therefore, both the VTA and SNc dopamine neurons may follow a unified encoding strategy for convergent processing of positive and negative motivational signals.

In the VTA, an earlier study has demonstrated that different populations of VTA putative dopamine neurons were activated or suppressed by differential fear conditioning [35]. Recently, it was reported that dopamine neurons located in ventral portion of the VTA were activated by footshocks in anesthetized rats [36]. However, these two studies did not examine how the same dopamine neurons would react to reward or positive events. By taking advantage of freely behaving states of our recording mice, we presented the mice with both positive and negative stimuli and found that vast majority of the VTA dopamine neurons react to reward and negative experiences.

It is important to note that our current extracellular recording technique does not have the capacity to visualize different types of putative dopamine neurons in our experiment. We estimate that type-3 dopaminergic-like neurons recorded in our experiment seem to be located more dorsally or anteriorly in the VTA area (Figure 1A, red and purple squares). However, it was noted that at least 12 pairs of type-1/2 and type-3 neurons were recorded simultaneously and in several cases recorded from one tetrode (e.g., Figure 3G; Figure 9J). Further more careful anatomic experiments may need to address this issue. Nonetheless, our results from awake freely behaving mice further support the notion that while the majority of the VTA putative dopamine neurons exhibit decreased activity, a small group of dopaminergic-like neurons can be activated by negative or aversive events. The type-3 dopaminergic-like neurons recorded in our experiment shared more similarity with type-1/2 putative dopamine neurons rather than non-dopamine neurons: all three types of neurons exhibited low baseline firing rate (0.5–10 Hz), relatively long inter-spike interval (>4 ms) and regular firing pattern. On the other hand, VTA non-dopamine neurons mostly exhibited higher baseline firing rate (>10 Hz) and strong modulation by movement [21]–[23]. In response to the two fearful events, the majority of these non-dopamine neurons (>70%) exhibited significant activation and with a great diversity of temporal firing patterns. The complex baseline activity, as well as response properties of these non-dopamine neurons to the two fearful events is beyond the scope of the discussion here.

Our current findings also provide several novel insights into the role of VTA dopamine neurons in both positive and negative motivation. First, VTA putative dopamine neurons respond to different negative stimuli in similar manners in awake animals. That is, neurons that responded to free fall always responded to shake in a similar manner (suppression for type-1 and type-2 neurons, activation for type-3 neurons). The uni-directional responses to the negative events within a given type of VTA dopamine neurons are similar to that of their responses to a wide range of novel and reward-related events [5], [37].

The second notable feature is the strong offset rebound excitation of type-1 dopamine neurons at the termination of free fall or shake events. This offset excitation in the freely behaving animals may encode information reflecting not only a relief at the termination of such fearful events [38]–[40], but perhaps providing some sorts of motivational signals (e.g., motivation to escape). It is also equally possible that the offset-redound excitation may play an important role in engaging thrill-seeking behaviors (e.g., extreme sports, Tower of Terror ride at Disney World). It is noteworthy to point out that the rebound activation of the VTA dopamine neuron was also reported at the termination of the footshock stimuli in anesthetized rats [36]. Nonetheless, it will be of great interest to further examine the dopamine neuron's functional relevance in various risky behaviors.

Third, VTA putative dopamine neurons exhibit temporal dynamic activities that correlate tightly with the durations of the fearful events. The use of temporal activity change for encoding the fearful event duration seems to make a good sense because that the suppression is very limited due to the low baseline firing rate of most dopamine neurons. This is interesting in compare with of the finding that dopamine neurons exhibit different peak responses to different values of reward boluses [41]. In considering the sources that drive the suppression of type-1 and type-2 dopamine neurons, recent studies suggest that the lateral habenular nucleus (LHb) and the GABAergic rostromedial tegmental nucleus (RMTg) play important roles [42]–[45]. First, these nuclei exhibit opposing responses to the rewarding or aversive stimuli compared with dopamine neuron's responses to the same stimuli [42], [44]. Second, dopamine neurons are strongly suppressed after activation of LHb or RMTg [43], [45].

Fourth, we further reveal that VTA dopamine neurons can exhibit completely opposite change in their firings by the conditioned stimulus for signaling either reward or fearful events which occurred under distinct contexts (Figure 6). This strongly suggests that neural processing occurring at the VTA level is highly integrated and the contextual information is an integral part of the encoding process for both positive and negative experiences. This finding is consistent with the anatomical evidence and previous hypotheses that the VTA neurons receive highly processed information from the forebrain structures such as hippocampus and prefrontal cortex [37], [46]–[48]. This high-level integration of experiences and events at the VTA neuronal population may explain why environments play such a dominating role in eliciting craving or reinforcement of habits.

Finally, our simultaneous recording techniques have allowed us to demonstrate a significant correlation among the type-1 and type-2 putative dopamine neurons, as well as among the type-3 neurons. The specificity of such firing synchrony is highly interesting, given the consideration of the possible VTA network arrangement. This suggests that VTA putative dopamine neurons may employ two highly specific synchronized strategies for optimizing and dopamine transmission efficacy and thereby providing a coordinated modulation of downstream structures such as nucleus accumbens. Lack of synchronized activity between type-3 and type-1/2 neurons is consistent with many other differences among them, both electro-physiologically and pharmacologically (Figure 3). Particularly, unlike type-1 and type-2 putative dopamine neurons, nearly all (96%; 23/24) of which exhibit significant suppression, type-3 neurons otherwise show excitation by the dopamine receptor agonists (Figure 3H). It is noted that putative dopamine neurons were reported to be mainly inhibited or not affected by dopamine receptor agonist in previous studies. Only a few studies have reported that some dopamine neurons can be activated by dopamine receptor agonists [24], [25], perhaps because the activated neurons were simply classified as non-dopamine neurons in previous studies. Notably, a small number of VTA dopamine neurons which are also TH-positive, have been reported to be activated by the dopamine receptor agonist [25]. Future experiments, perhaps using optogenetics, will be required to confirm whether those fear-activated type-3 neurons were dopamine neurons. And the acceptance of these type-3 neurons as dopamine neurons should be with caution to date.

In summary, we show that the vast majority of VTA putative dopamine neurons are capable of responding to both reward and fear-driven aversive information. These putative dopamine neurons respond to different negative events in a similar manner and more importantly, their temporal durations of dynamic firing changes are proportional to the durations of the fearful events. VTA putative dopamine neurons also integrate cues and contextual information for distinction between reward and fearful events. Taken together, we suggest that VTA dopamine neurons may employ the convergent encoding strategy at the network population level for processing both positive and negative experiences. Such convergent encoding of experiences is also highly integrated with cues and environmental contexts to further enhance behavioral specificity.

## Materials and Methods

### Ethics statement

All animals used in this study were according to procedures approved by the Institutional Animal Care and Use Committee, Georgia Health Sciences University and covered under protocol number BR-07-11-001.

### Subjects

A total of 71 male C57BL/6J mice were used for recording and individually housed on a 12-h light/12-h dark cycle. Only data from 24 mice from which we recorded putative dopamine neurons were used in the current analyses.

### Surgeries

A 32-channel (a bundle of 8 tetrodes), ultra-light (weight <1 g), movable (screw-driven) electrode array was constructed similar to that described previously [49]. Each tetrode consisted of four 13-µm diameter Fe-Ni-Cr wires (Stablohm 675, California Fine Wire; with impedances of typically 2–4 MΩ for each wire) or 17-µm diameter Platinum wires (90% Platinum 10% Iridium, California Fine Wire; with impedances of typically 1–2 MΩ for each wire). One week before surgery, mice (3–6 months old) were removed from the standard cage and housed in customized homecages (40×20×25 cm). On the day of surgery, mice were anesthetized with Ketamine/Xylazine (80/12 mg/kg, i.p.); the electrode array was then implanted toward the VTA in the right hemisphere (3.4 mm posterior to bregma, 0.5 mm lateral and 3.8–4.0 mm ventral to the brain surface) and secured with dental cement.

### Tetrode Recording and Units Isolation

Two or three days after surgery, electrodes were screened daily for neural activity. If no dopamine neurons were detected, the electrode array was advanced 40∼100 µm daily, until we could record from a putative dopamine neuron. Multi-channel extracellular recording was similar to that described previously [49]. In brief, spikes (filtered at 250–8000 Hz; digitized at 40 kHz) were recorded during the whole experimental process using the Plexon multichannel acquisition processor system (Plexon Inc.). Mice behaviors were simultaneously recorded using the Plexon CinePlex tracking system. Recorded spikes were isolated using the Plexon OfflineSorter software: multiple spike sorting parameters (e.g., principle component analysis, energy analysis) were used for the best isolation of the tetrode-recorded spike waveforms. Combining the stability of multi-tetrode recording and multiple unit-isolation techniques available in OfflineSorter (e.g., principle component analysis, energy analysis), individual VTA neurons can be studied in great detail, in many cases for days (Figure S1).

### Fearful Events

Two fearful events, free fall (from 10 and 30 cm) and shake (for 0.2, 0.5 and 1 sec), were randomly performed in our experiments with an interval of typically 1–2 hours between sessions. We used either a square (10×10×15 cm) or round chamber (11 cm in diameter, 15 cm in height) for the free fall event. We used a round chamber (12.5 cm in diameter, 15 cm in height) for the shake events. In each free fall or shake event session, a mouse was placed into the free fall or shake chamber (mouse could move freely inside the chambers). After 3 min habituation, about 20 trials of free fall (or shake) events were given with an interval of 1–2 min between trials. The free fall chamber was lifted up (either 10 cm or 30 cm height) and tied to a solenoid system (Magnetic Sensor Systems, Series S-20-125) before each free fall event. The free fall event was then delivered by providing precise mechanical control (WPI, PulseMaster A300) of the solenoid system to release the suspension rope. The free fall chamber then landed on a soft pad that greatly reduced the bounces and prevented potential damages to the stability of the recording (Figures S2 and S3). Free fall duration was calculated by the equation: T =  SQRT (2×h/g), where h is the height of free fall, and g is the acceleration of the earth's gravity. Considering the soft landing delay, the estimated durations for 10 and 30 cm free falls were 230 and 340 ms, respectively. The shake event was delivered by providing precise mechanical control of a vortex machine (Thermolyne Maxi Mix II Type 37600 Mixer) at a maximum speed of 3000 rpm throughout unless for the low-intensity one, which was about 1500 rpm.

We always monitored the stability of recorded units by examining the spike waveforms, baseline firing status, and spike cluster distributions before and after the events as well as through the entire experiments. We only included the datasets from the animals that met these recording criteria for further data analyses. As shown in Figures S1, S2, and S3, dopamine neurons listed in the present study were stably recorded and well isolated during both free fall and shake events, without temporary loss of unit or noise/artifact contamination.

In particular, we took three steps to ensure that spikes were not contaminated by any artifacts: 1) We reduced the interference for the recording by grounding the whole experimental apparatus. We found that the electrical artifacts generated during the free fall and shake events were at the similar level of those during locomotor exploration. 2) We further cancelled out the remaining artifacts by the Plexon Referencing Client which allowed us to choose a channel with no visibly good units as a reference channel. This greatly eliminated background noises and artifacts. 3) If any possible artifact waveforms were still left, we then removed them during the preprocessing of spike waveforms using Plexon Offline Sorter because artifact waveforms were highly distinct from neuronal spike waveforms.

### Reward and Bi-directional Conditioning

Mice were slightly food restricted before reward association training. In reward conditioning, mice were placed in the reward chamber (45 cm in diameter, 40 cm in height). Mice were trained to pair a tone (5 kHz, 1 sec) with subsequent sugar pellet delivery for at least two days (40–60 trials per day; with an interval of 1–2 min between trials). The tone was generated by the A12-33 audio signal generator (5-ms shaped rise and fall; about 80 dB at the center of the chamber) (Coulbourn Instruments). A sugar pellet (14 mg) was delivered by a food dispenser (ENV-203-14P, Med. Associates Inc.) and dropped into one of two receptacles (12×7×3 cm) at the termination of the tone (the other receptacle was used as control, where a sugar pellet was never received).

In a separate set of experiments, mice were trained for bi-directional conditioning (both reward and aversive conditioning). The conditioned tone (5 kHz, 1 sec) used was identical, but in different contexts: during reward conditioning (in the reward chamber; 45 cm in diameter, 40 cm high), the tone was paired with sugar pellet delivery; during aversive conditioning (in the free fall chamber), the same tone was paired with a free fall event (30 cm high). Mice were trained for one week or more and were counterbalanced: half of the mice received reward conditioning on days 1 and 2, followed by aversive conditioning on days 3 and 4 (40–60 trials each day); the other half of the mice received aversive conditioning on days 1 and 2, followed by reward conditioning on days 3 and 4 (40–60 trials per day). On days 5 and later, three sessions (20–30 trials per session) were given each day in a random order, including reward conditioning, aversive conditioning, and in a third neutral chamber (55×30×30 cm that was enriched with toys) where the tone did not predict anything. The interval between sessions was 1–2 hours; the interval between trials was 1–2 min. The latency of sugar/control receptacle approach after onset the conditioned tone was examined on day 7. Latencies longer than 60 sec were considered as 60 sec; in the case that the mouse was inside the receptacle during the conditioned tone, the latency was not used for calculation. The backward movement behavior (head and/or limbs moving backward) after onset the conditioned tone was also examined on day 7.

### Histological Verification of Recording Site

On completion of the experiments, the final electrode position was marked by passing a 10-sec, 20-µA current (Stimulus Isolator A365, WPI) through two electrodes. Mice were deep anesthetized and perfused with 0.9% saline followed by 4% paraformaldehyde. Brains were then removed and post-fixed in paraformaldehyde for at least 24 h. Brains were rapidly frozen and sliced on a cryostat (50-µm coronal sections) and stained with cresyl violet. The histological experiments were performed on 21 mice (in another 3 mice the brain sections were unfortunately not well prepared). Our histology results confirmed that dopamine neurons were recorded from the VTA area in 17 mice and from the VTA-SNc border area in 4 mice (Figure 1A).

### Data Analysis

Sorted neural spikes were processed and analyzed in NeuroExplorer (Nex Technologies) and Matlab. Dopamine neurons were classified based on the following three criteria: 1) low baseline firing rate (0.5–10 Hz); 2) relatively long inter-spike interval (all the classified putative dopamine neurons are with ISIs >4 ms within a ≥99.8% confidence level). The shortest ISI we recorded was 4.1 ms under any conditions in our experiment (only well-isolated units with amplitude ≥0.4 mV were used for calculation of the shortest ISI). The averaged shortest ISIs was 6.8±2.2 ms (Mean ± s.d.; n = 36). In contrast, the ISI for non-dopamine neurons can be as short as 1.1 ms; 3) regular firing pattern when mice were freely behaving (fluctuation <3 Hz). Here, fluctuation represents the standard deviation (s.d.) of the firing rate histogram bar values (bin  = 1 sec; recorded for at least 600 sec). In addition, it was noted that the vast majority (89%; 56/63) of the classified dopamine neurons tested showed significant activation in response to the reward predicting tone (Figure 2E and F). It was also noted that the majority of the classified putative dopamine neurons (70%, 23/33; type-1 and 2) tested showed significant suppression (≤30% baseline firing rate) and the other 27% type-3 neurons (n = 9) showed activation (Figure 3H). On the other hand, VTA non-dopamine neurons showed limited or no change in firing rate by the dopamine receptor agonists (Figure 3I). Half AP widths of the spike waveforms were measured from the troughs to the following peaks of the action potential (Figure 1B). Half AP widths broader than 0.8 ms were considered as 0.8 ms. For the calculation of dopamine neuron's burst firing probability, baseline activity when mice were free behaving was used according to previous established criteria (burst onset, ISI of ≤80 ms; burst offset, ISI of ≥160 ms) [50].

Neuronal activity changes to the conditioned and unconditioned stimuli were compared against a 10-sec control period before the onset of the stimulus in each trial with a chosen time window (depending on the durations of the stimuli) using a Wilcoxon signed-rank test. For 10 and 30 cm free fall events, the time windows were 100–230 and 100–340 ms after onset of the free fall event, respectively; for 0.2, 0.5 and 1 sec shake events, the time windows were 100–200, 100–500, and 100–1000 ms after onset of the shake event, respectively (it was noted that a few type-1/2 putative dopamine neurons, ∼10%, also showed a small activation during the initial 100 ms right after onset of the free fall and shake events). For reward conditioning, the time window was 50–600 ms after onset of the conditioned tone; for aversive conditioning, the time window was 200–600 ms after onset of the conditioned tone.

Peri-event rasters (1–20 trials, from top to bottom) and histograms were conducted in NeuroExplorer (Nex Technologies). All smoothings were conducted in the NeuroExplorer using a Gaussian filter (filter width  = 3 bins). Cross-correlations were conducted between simultaneously recorded dopamine neuron pairs when mice were freely behaving (without external stimuli) or sleeping in the homecage. For z-score calculation of the cross-correlation peak value, the cross-correlation histograms were smoothed to get the peak value; mean and standard deviations were obtained from shuffled (randomized) spikes in Matlab [51]. It is noted that the synchronized units represent different dopamine neurons rather than the same neuron. We ruled out the possibility that the synchronized units were recorded from or contaminated by the same neuron (when it happened, there would be a sharp peak at a time widow of ∼1 ms instead of ∼100 ms as showed in Figure 9).

## Supporting Information

Figure S1 VTA dopamine neurons are stably recorded and well isolated. (A) An example of a well-isolated type-1 dopamine neuron (blue dots) in a 2-dimensional Principal Component Analysis and its representative waveforms (recorded by tetrode) on day 1 (upper panel) and day 2 (lower panel). Spike isolation was performed using Plexon OfflineSorter (Plexon Inc. Dallas, TX). PC1 and PC2 represent the first and second principal components, respectively. Blue dots represent individual spikes for the isolated dopamine neuron; black dots indicate individual spikes for other VTA neurons. (B) An example of a well-isolated type-2 dopamine neuron (blue dots) and its representative waveforms on day 1 (upper panel) and day 2 (lower panel). (C) An example of a well-isolated type-3 dopamine neuron (blue dots) and its representative waveforms on day 1 (upper panel) and day 2 (lower panel).
(TIF)

Figure S2 No temporary loss of unit during free fall and shake events. (A) Responses of four simultaneously recorded VTA dopamine and non-dopamine neurons during free fall events. Note that units recorded from the same tetrode may exhibit opposite responses (e.g., tetrode #5 units 1 & 2; tetrode #8 units 1 & 2), suggesting that the recording was stable without any temporary losses of units. (B) Responses of the same four VTA neurons during shake events. (C) Representative waveforms for the same four VTA neurons 1 h before, during free fall and shake event session, and 1 h after.
(TIF)

Figure S3 No noise/artifact contamination during free fall and shake events. (A) Responses of an example putative dopamine neuron (type-1) and its waveforms before (1 sec), during (1 sec), and after (1 sec) the free fall and shake events. Note that the waveforms did not show significant change after free fall and shake event, suggesting that there was no noise/artifact contamination. (B) Responses of another putative dopamine neuron (type-3) and its waveforms before (1 sec), during (1 sec), and after (1 sec) free fall and shake events.
(TIF)
