# Supplementary figures and images for: Convergent Processing of Both Positive and Negative Motivational Signals by the VTA Dopamine Neuronal Populations
Source: PLoS One. 2011 Feb 15;6(2):e17047. doi: 10.1371/journal.pone.0017047 (PMC3039659; doi:10.1371/journal.pone.0017047)

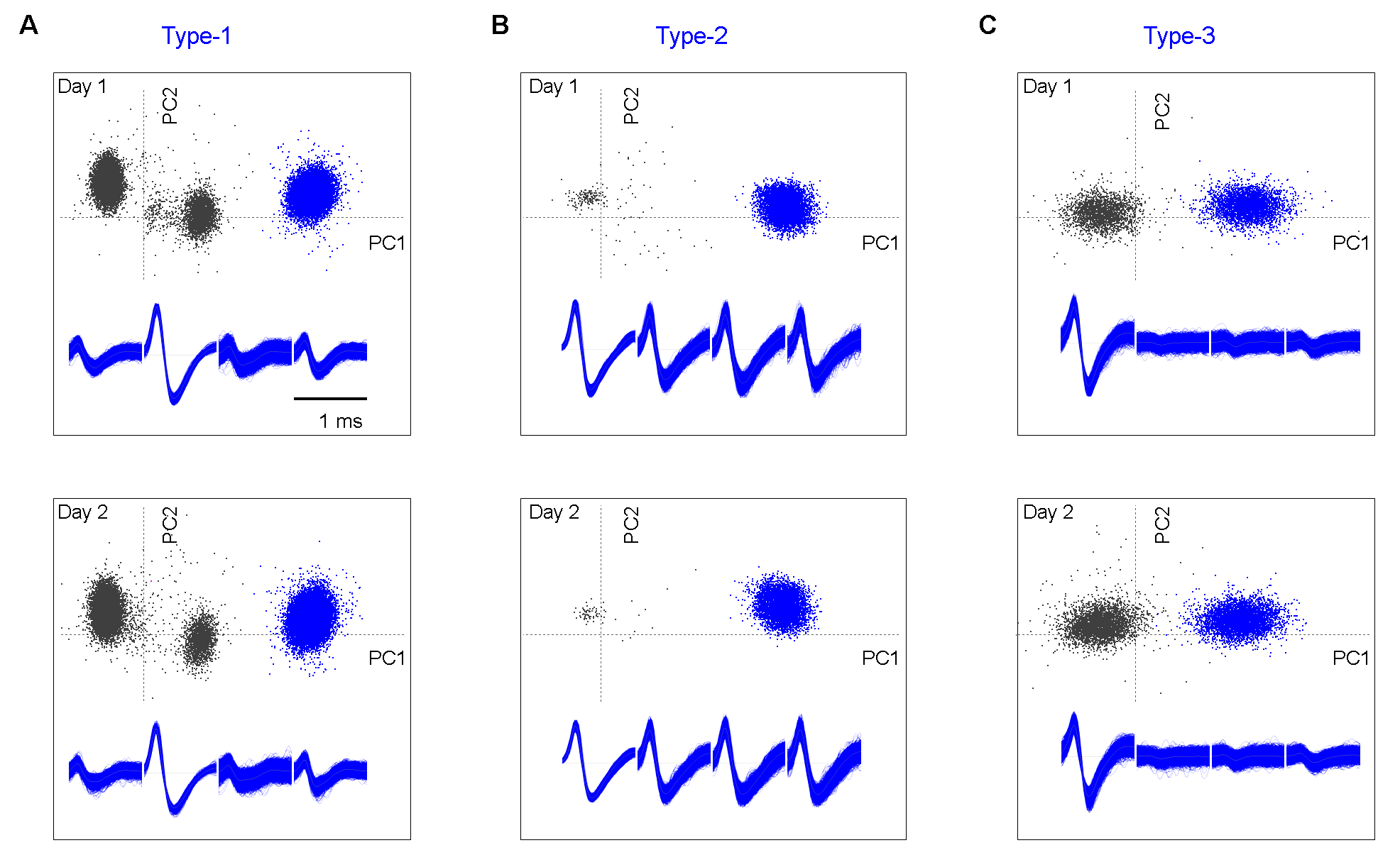

Supplement: Figure S1 — VTA dopamine neurons are stably recorded and well isolated. (A) An example of a well-isolated type-1 dopamine neuron (blue dots) in a 2-dimensional Principal Component Analysis and its representative waveforms (recorded by tetrode) on day 1 (upper panel) and day 2 (lower panel). Spike isolation was performed using Plexon OfflineSorter (Plexon Inc. Dallas, TX). PC1 and PC2 represent the first and second principal components, respectively. Blue dots represent individual spikes for the isolated dopamine neuron; black dots indicate individual spikes for other VTA neurons. (B) An example of a well-isolated type-2 dopamine neuron (blue dots) and its representative waveforms on day 1 (upper panel) and day 2 (lower panel). (C) An example of a well-isolated type-3 dopamine neuron (blue dots) and its representative waveforms on day 1 (upper panel) and day 2 (lower panel). (TIF) [file pone.0017047.s001.tif]

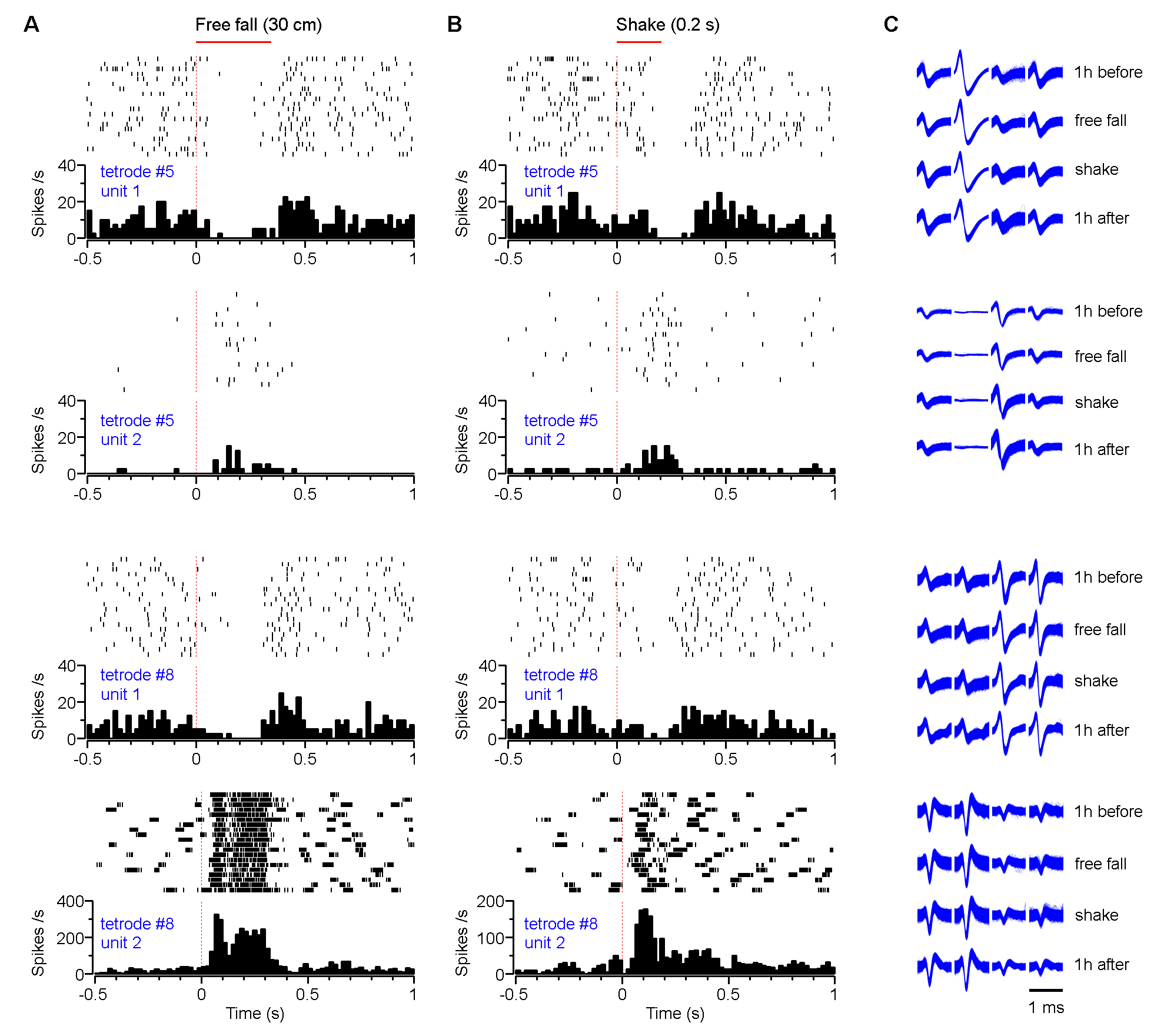

Supplement: Figure S2 — No temporary loss of unit during free fall and shake events. (A) Responses of four simultaneously recorded VTA dopamine and non-dopamine neurons during free fall events. Note that units recorded from the same tetrode may exhibit opposite responses (e.g., tetrode #5 units 1 & 2; tetrode #8 units 1 & 2), suggesting that the recording was stable without any temporary losses of units. (B) Responses of the same four VTA neurons during shake events. (C) Representative waveforms for the same four VTA neurons 1 h before, during free fall and shake event session, and 1 h after. (TIF) [file pone.0017047.s002.tif]

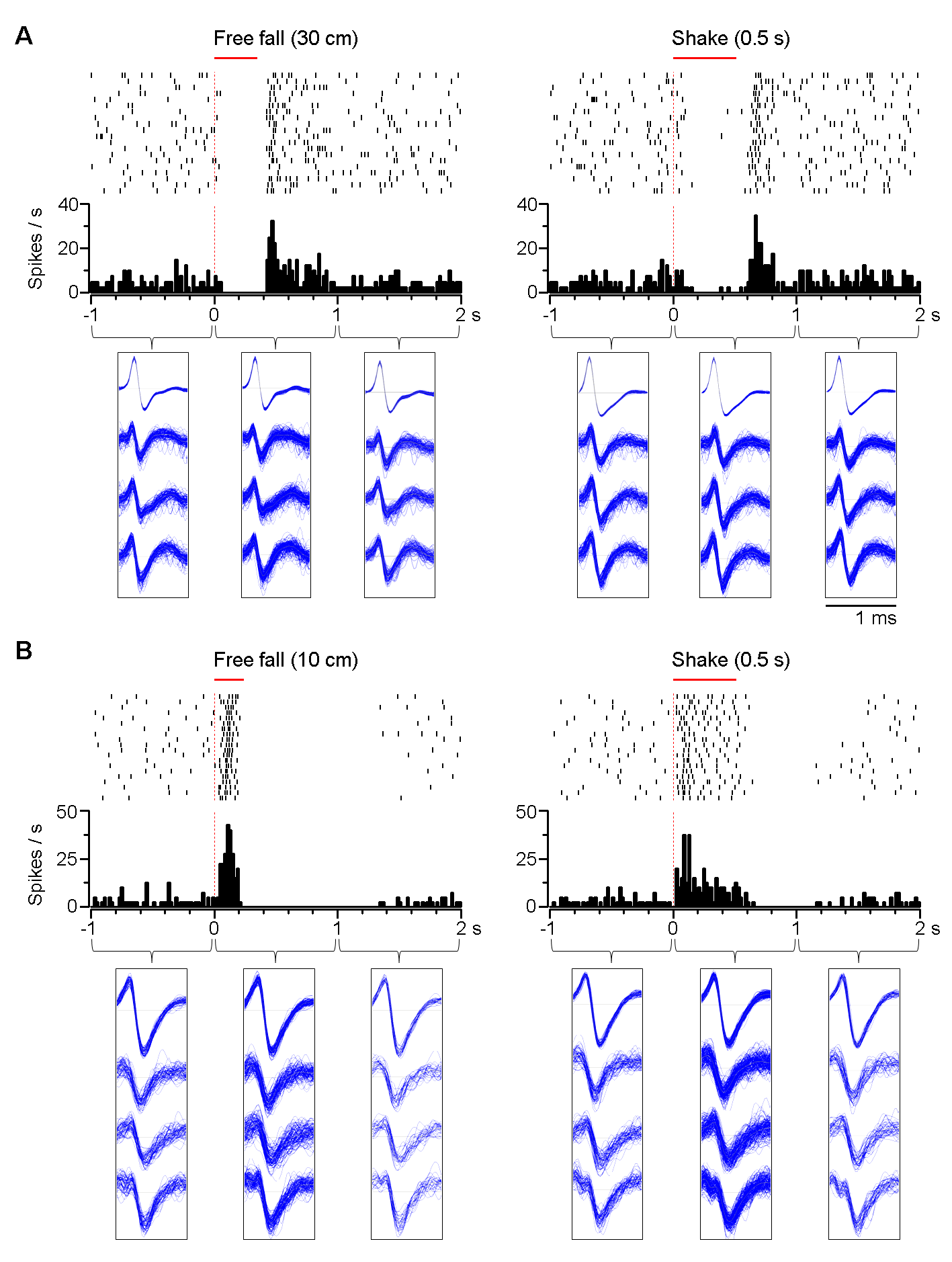

Supplement: Figure S3 — No noise/artifact contamination during free fall and shake events. (A) Responses of an example putative dopamine neuron (type-1) and its waveforms before (1 sec), during (1 sec), and after (1 sec) the free fall and shake events. Note that the waveforms did not show significant change after free fall and shake event, suggesting that there was no noise/artifact contamination. (B) Responses of another putative dopamine neuron (type-3) and its waveforms before (1 sec), during (1 sec), and after (1 sec) free fall and shake events. (TIF) [file pone.0017047.s003.tif]
